# Evaluation of the response of creole cows (*Bos taurus*) to two fixed-time artificial insemination protocols in the High-Andean region of Ayacucho, Peru

**DOI:** 10.29374/2527-2179.bjvm000326

**Published:** 2026-09-30

**Authors:** Mijaíl Contreras Huamaní, César Jorge Mendoza Leiva, Hamilton Guzman Santaria, Walter Palomino-Guerrera, Hurley Abel Quispe-Ccasa

**Affiliations:** 1 Estación Experimental Agraria Canaán, Proyecto PROGAN, Instituto Nacional de Innovación Agraria, Ayacucho, Peru.; 2 Dirección de Investigación y Desarrollo Tecnológico, Proyecto PROGAN, Instituto Nacional de Innovación Agraria, Lima, Peru.

**Keywords:** high-Andean livestock farming, estrus synchronization, estradiol cypionate, corpus luteum, reproductive efficiency, pecuária de alta montanha, sincronização de cio, cipionato de estradiol, corpo lúteo, eficiência reprodutiva

## Abstract

Unlike specialized breeds, High-Andean Creole cattle have developed a remarkable adaptability and hardiness to challenging high-altitude conditions, albeit at the expense of productivity. However, the efficiency of their reproductive performance under estrus synchronization protocols for fixed-time artificial insemination (FTAI) remains scarcely addressed. This study aimed to evaluate the response of Creole cows to two FTAI protocols in the districts of Chuschi (Cangallo) and Chipao (Lucanas), Ayacucho, Peru. From a sample of 60 Creole cows (identified by phenotype and coat color), with uniform body condition scores and management system, 35 were synchronized using a three-handling protocol (T1: estradiol cypionate and Bovine Intravaginal device (BID) removal on day 8), and 25 using a four-handling protocol (T2: BID removal on day 7 and estradiol benzoate on day 8). The cows were inseminated using thawed semen from Fleckvieh and Brown Swiss bulls, the latter belonging to the genetic nucleus of the Instituto Nacional de Innovación Agraria. Estrus detection rates ranged from 88.6% to 80.0%, yielding pregnancy rates of 57.1% and 56.0% for T1 and T2, respectively (p>0.05). No significant effects of protocol, age, number of calvings, body condition score, or bull breed were found on estrus detection, pregnancy, or calf sex (p>0.05). However, cows with a corpus luteum had 7.1-fold higher odds of pregnancy than those presenting only follicles (p<0.05). In conclusion, both FTAI protocols demonstrated comparable reproductive performance; however, the three-handling protocol with estradiol cypionate emerges as the preferred option due to its lower cost and greater operational simplicity under challenging High-Andean conditions.

## Resumo

O gado Crioulo de Alta Montanha desenvolveu forte adaptabilidade e rusticidade às condições desafiadoras de grande altitude, ao contrário das raças especializadas, embora em detrimento da sua produtividade. No entanto, a eficiência do seu desempenho reprodutivo sob protocolos de sincronização de cio para inseminação artificial em tempo fixo (IATF) ainda é pouco abordada. Esta pesquisa teve como objetivo avaliar a resposta de vacas Crioulas a dois protocolos de IATF nos distritos de Chuschi (Cangallo) e Chipao (Lucanas), Ayacucho, Peru. A partir de uma amostra de 60 vacas Crioulas (identificadas por fenótipo e pelagem), com escore de condição corporal e sistema de manejo uniformes, 35 foram sincronizadas utilizando um protocolo de três manejos (T1: cipionato de estradiol e retirada do dispositivo intravaginal de progesterona [BID] no dia 8), e 25 utilizando um protocolo de quatro manejos (T2: retirada do BID no dia 7 e benzoato de estradiol no dia 8). As vacas foram inseminadas com sêmen descongelado de touros das raças Fleckvieh e Brown Swiss, esta última pertencente ao núcleo genético do Instituto Nacional de Innovación Agraria. As taxas de detecção de cio variaram de 88,6% a 80,0%, resultando em taxas de prenhez de 57,1% e 56,0% para T1 e T2, respectivamente (p>0,05). Não foram encontrados efeitos significativos do protocolo, idade, número de partos, escore de condição corporal ou raça do touro sobre a detecção de cio, prenhez ou sexo do bezerro (p>0,05). No entanto, vacas com corpo lúteo apresentaram uma chance 7,1 vezes maior de prenhez do que aquelas que apresentavam apenas folículos (p<0,05). Em conclusão, ambos os protocolos de IATF demostraram desempenho reprodutivo comparável; contudo, o protocolo de três manejos com cipionato de estradiol surge como a opção preferencial devido ao seu menor custo e maior simplicidade operacional sob as desafiadoras condições da região de Alta Montanha.

## Introduction

Research in Creole cattle (*Bos taurus*) is highly relevant as these animals represent a valuable genetic resource characterized by rusticity, adaptation to harsh environments, disease resistance, and the efficient utilization of scarce forage resources (Pérez-Ruiz et al., 2022). In the High-Andean region, livestock production relies primarily on natural pastures whose availability fluctuates markedly with seasonal rainfall, alongside challenges such as an arid climate, low oxygen availability, low temperatures, and high solar radiation. The environmental variability likely influences reproductive success through its direct impact on nutrition (Delgado et al., 2019; Mastromonaco & Gonzalez-Grajales, 2020).

Estrus synchronization and artificial insemination have become indispensable tools for driving genetic improvement across all scales of cattle production. When adequate infrastructure and trained personnel are lacking, Fixed-Time Artificial Insemination (FTAI) is commonly implemented. Estrus synchronization protocols rely on exogenous hormonal combinations that synchronize both the emergence of a new follicular wave and ovulation, thereby enabling artificial insemination without the need for estrus detection (Kasimanickam, 2021; Pal & Rayees Dar, 2021; Pérez-Ruiz, et al., 2022). Furthermore, FTAI increases service rates by allowing the inclusion of cows regardless of their ovarian cyclicity status (Salgado-Otero et al., 2015); consequently, it is widely used in intensive and semi-intensive systems, though to a lesser extent in extensive systems.

Synchronization protocols using progestogens involve the insertion of a Bovine Intravaginal Device (BID) loaded with slow-release progesterone, which maintains the luteal phase until its removal, after which the follicular phase is reinitiated. This approach was designed to increase pregnancy rates, facilitate estrus detection, and support embryo collection and transfer programs, as well as anestrus treatments. Progesterone is produced naturally by the ovaries; its presence prevents complete follicular maturation and ovulation, whereas a decrease in its concentration triggers follicular growth (La Marca & Capuzzo, 2019; Pal & Rayees Dar, 2021). This device is complemented by the administration of Prostaglandin (PGF2α) and Equine Chorionic Gonadotropin (eCG). Prostaglandin (PGF2α) accelerates luteolysis, while eCG exerts a follicle-stimulating effect, thereby inducing and synchronizing estrus. Additionally, the protocol incorporates exogenous estradiol, which reduces the frequency of animal handlings, facilitates control of follicular dynamics and ovulation induction, optimizes FTAI scheduling, and decreases handling-related stress (Kumar et al., 2014; Pal & Rayees Dar, 2021).

In Creole cattle from High-Andean regions, reports on the efficacy of estrus synchronization and FTAI protocols remain scarce, although stable reproductive performance between the dry and rainy seasons has been described (Alfaro-Astorima et al., 2020). However, slight variations in follicular dynamics have been reported compared to standard bovine parameters, including up to three follicular waves per estrous cycle (Alfaro-Astorima et al., 2020), which could complicate the direct extrapolation of standard FTAI protocols (Mastromonaco & Gonzalez-Grajales, 2020), and suggest the need for specific adaptations. Furthermore, there is a critical need to simplify these protocols due to the logistical and accessibility constraints of remote regions, without compromising reproductive efficiency. Therefore, the objective of this study was to evaluate the response of Creole cows to two FTAI protocols in the High-Andean region of Ayacucho, Peru.

## Material and methods

The study was conducted from February to July 2024 on cattle farms located in the district of Chuschi, Cangallo Province (13°30′03″ S, 74°08′28″ W), and in the district of Chipao, Lucanas Province (14°35’00” S, 74°15’00” W), Ayacucho, Peru, at altitudes ranging between 3,900 and 4,000 meters above sea level. The study area is characterized by an arid climate with average annual temperatures between 5 and 12 °C, marked diurnal temperature variations, and a seasonal rainfall pattern concentrated between November and March, yielding an annual precipitation of approximately 600 to 800 mm.

### Experimental design

The study followed a completely randomized unbalanced design to evaluate two estrus synchronization protocols applied to 60 Creole cows, which identified by phenotype and coat color according to Rojas-Espinoza et al. (2023). All cows were managed under similar breeding systems, environmental conditions, and feeding regimes based on natural pastures, with free access to iodized salt and water *ad libitum*. The cows were assigned to two treatments groups: 35 cows received the protocol T1, and 25 cows received the protocol T2. The protocols are outlined in Figure 1.

**Figure 1:**
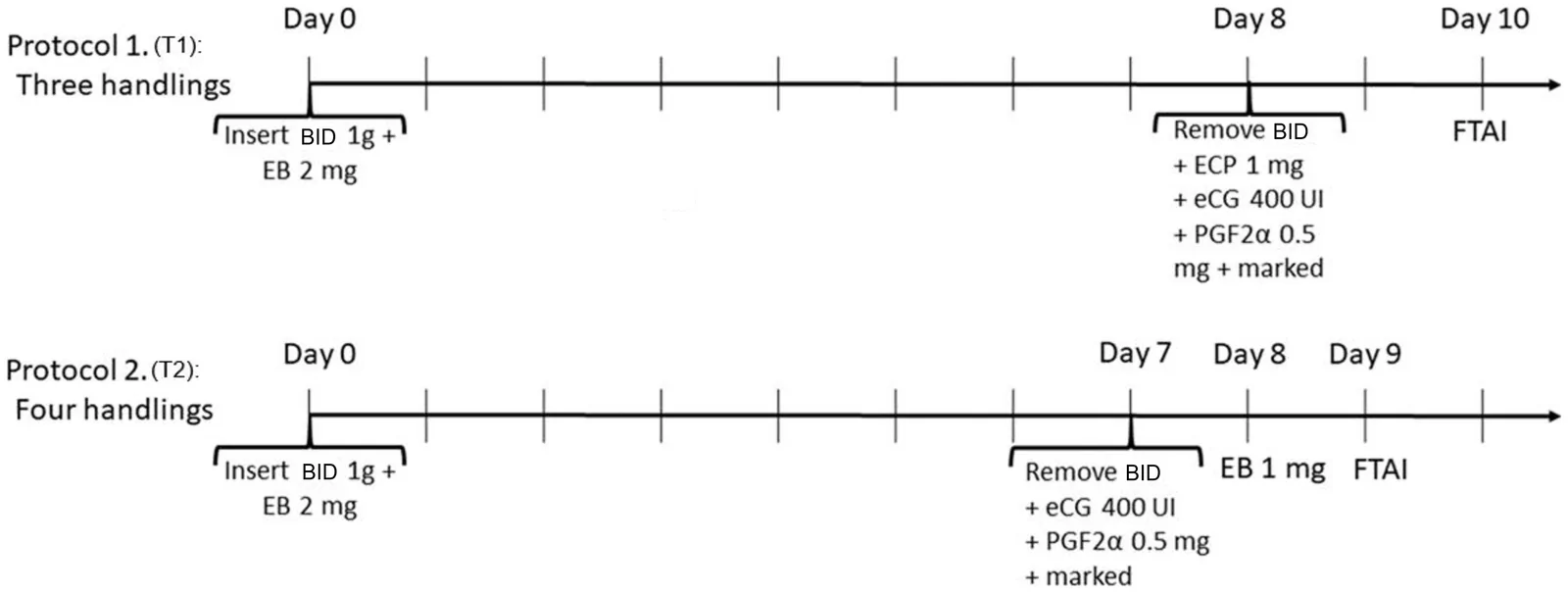
Experimental protocols for estrus synchronization for fixed-time artificial insemination (FTAI) in Creole cows from the High-Andean region of Ayacucho. BID = Bovine Intravaginal Device; EB = Estradiol Benzoate; ECP = Estradiol Cypionate; eCG = Equine Chorionic Gonadotropin; PGF2α = Prostaglandin.

### Selection and gynecological evaluation

To maximize pregnancy probability, healthy primiparous and multiparous cows alder than three years with a body condition score between (BCS) between 2.5 and 3.0 (on a 1-5 scale) were selected. Functional ovaries, including the presence of follicles and corpora lutea, were confirmed via ultrasonography using a 7.5 MHz linear transducer (Esaote Tringa Linear VET, Holland). Following selection, cows were dewormed with Albendazole (1 mL/10 kg) and supplemented with 2-5 mL of vitamins A, D, and E (Vigantol®) and 10-20 mL of vitamin B12 (Catosal®) intramuscularly, adjusted by body weight.

### Estrus synchronization protocols

All hormones used for estrus synchronization were manufactured by Ourofino© (Brazil). The two protocols were administered as follows:

#### Protocol T1 (three handling)

– Day 0: Insertion of a slow-release progesterone Bovine Intravaginal Device (BID; 1 g progesterone, Sincrogest®) + 2 mg Estradiol Benzoate (Sincrodiol®), IM.
– Day 8: BID removal (Sincrogest®) + administration of 0.5 mg Prostaglandin PGF2α (Sincrocio®) + 400 IU eCG (Sincro eCG®) + 1 mg Estradiol Cypionate (SincroCP®), IM.
– Day 10: Fixed-time artificial insemination performed 48 hours after BID removal.

#### Protocol T2 (four handlings)

– Day 0: Insertion of a BID containing 1 g progesterone + 2 mg Estradiol Benzoate (Sincrodiol®), IM.
– Day 7: BID removal + administration of 0.5 mg PGF2α + 400 IU eCG, IM.
– Day 8: Administration of 1 mg Estradiol Benzoate, IM.
– Day 9: Fixed-time artificial insemination performed 52-56 hours after BID removal.

In all synchronized cows, estrus signs were recorded by a single trained technician from the time of BID removal until FTAI. Signs included standing heat (being mounting by other cows) and external indicators such as a wet, licked, disheveled, or abraded rump. Artificial insemination was performed using 0.5 mL frozen semen straws (20 x 10^6^ sperm/mL) from Brown Swiss bulls (from the genetic nucleus of the Instituto Nacional de Innovación Agraria) and Fleckvieh bulls, with post-thaw sperm motility exceeding 40%.

### Pregnancy diagnosis and reproductive efficiency

Pregnancy diagnosis was conducted 35 days after artificial insemination using an ultrasound scanner (Esaote Tringa Linear VET, Holland), and confirmed by the visualization of an embryonic heartbeat. Reproductive efficiency parameters included the estrus detection rate, calculated as the number of cows detected in estrus divided by the total number of synchronized cows ×100, and the pregnancy rate, calculated as the number of pregnant cows at 30 days post-insemination divided by the total number of inseminated cows ×100 (Salgado-Otero et al., 2015). Additionally, estimated costs per inseminated cow and per pregnant cow were compared, and calf sex was recorded at birth.

### Statistical analysis

Data were compiled in Excel spreadsheets and analyzed using SPSS v.15.0. Frequency tables and percentages were generated based on estrus synchronization protocol, district, age, number of calvings, body condition score, presence of corpus luteum, and bull breed. Associations between categorical variables and response variables (estrus detection rate, pregnancy rate, and calf sex) were analyzed using the χ^2^ test (p<0.05) when expected frequencies were greater than 5, and Fisher’s exact test for 2×2 contingency tables (p<0.05). Binomial correlations were assessed using Cramer’s V (p<0.05). The effects of categorical variables associated with estrus and pregnancy success were analyzed using logistic regression. The regression coefficients were used to calculate the odds ratios (OR) with their respective 95% confidence intervals to determine their contribution to estrus detection and pregnancy; these were plotted using R software v.4.4.3 (R Development Core Team, 2025).

## Results

The application of both protocols successfully synchronized a new follicular wave and ovulation, resulting in comparable pregnancy rates via FTAI. As shown in Table 1, the estrus detection and pregnancy rates were 88.6% and 57.1% for T1 (three handlings with estradiol cypionate and BID removal on day 8), and 80.0% and 56.0% for T2 (four handlings with estradiol benzoate and BID removal on day 7), respectively, with no significant differences between treatments (p>0.05). Furthermore, no significant association was found between calf sex ratios (female vs. male) and the synchronization protocols applied (p>0.05).

**Table 1:** Estrus detection rate, pregnancy rate, and calf sex in Creole cows from the High-Andean region subjected to two Fixed-Time Artificial Insemination (FTAI) protocols.

**FTAI Protocols**	**n**	**Estrus detection rate (%)**	**Pregnancy rate (%)**	**Female calves (%)**	**Male calves (%)**
T1 (three-handling)	35	88.6	57.1	44.4	55.6
T2 (four-handling)	25	80.0	56.0	50.0	50.0
p-value		0.47	0.93	1.00	

The associations between estrus detection success, pregnancy, and calf sex according to the FTAI protocol were analyzed using a χ2 independence test (p<0.05).

According to Table 2, categorical variables, including district, age, number of calvings, body condition score, and bull breed, were not associated with any reproductive efficiency parameters; estrus detection rates, pregnancy rates, and the percentages of male and female calves remained statistically similar across most groups (p>0.05). However, during the gynecological evaluation performed prior to initiating the FTAI protocols, the presence of a corpus luteum was associated with a higher pregnancy rate (75.0%) compared with cows presenting only follicles (42.1%) (p<0.05). This ovarian characteristic was not associated with estrus detection rate or calf sex (p>0.05).

**Table 2:** Association between categorical variables and reproductive efficiency variables in Creole cows from the High-Andean region subjected to two FTAI protocols.

**Categorical variables**	**Levels**	**n**	**Estrus detection rate (%)**	**Pregnancy rate (%)**	**Female calves (%)**	**Male calves (%)**
District	Chuschi	35	85.7	56.0	40.0	60.0
	Chipao	25	84.0	57.1	50.0	50.0
	p-value		1.00	0.93	0.62	
Age	3-4 years-old	23	95.7	56.5	50.0	50.0
	> 5 years-old	37	78.4	56.8	43.8	56.3
	p-value		0.13	0.99	0.76	
Number of calving	0-1 calving	25	92.0	56.0	38.5	61.5
	>2 calvings	35	80.0	57.1	53.8	46.2
	p-value		0.28	0.93	0.43	
BSC	<2.50	5	81.8	63.6	40.0	60.0
	2.75	15	80.6	58.1	46.7	53.3
	>3.00	6	94.4	50.0	50.0	50.0
	p-value		0.41	0.75	0.95	
Corpus luteum	Absent	16	84.2	42.1	42.9	57.1
	Presence	18	90.0	75.0	50.0	50.0
	p-value		0.66	0.04	0.76	
Straws breed	Brown Swiss	46	84.8	60.9	50.0	50.0
	Fleckvieh	14	85.7	42.9	25.0	75.0
	p-value		1.00	0.23	0.36	

The associations between estrus detection success, pregnancy, and calf sex according to the categorical variables were analyzed using a χ^2^ independence test (p<0.05). BSC = Body Score Condition.

Analysis of the association between the primary response variables revealed a non-significant dependency and weak binary correlation (Fisher’s exact test = 0.157; R = 0.198; Cramer’s V = 0.125). Of the 51 cows with positive estrus detection, only 31 resulted in confirmed pregnancies. Furthermore, out of 34 total confirmed pregnancies, estrus was detected in 31 cases (91.2%), whereas among the 26 non-pregnant cows, estrus was detected in 20 (76.9%).

As shown in Table 3, the logistic regression analysis of estrus detection and pregnancy rate indicated that Protocol T1 achieved a slight efficiency advantage over Protocol T2, though this difference was not statistically significant (p>0.05). A similar non-significant pattern was observed when comparing cows with low parity (0-1 calvings) to those with more than two calvings, as well as when comparing Brown Swiss versus Fleckvieh semen. The effects of age and body condition score were likewise non-significant (p>0.05). Conversely, cows with a corpus luteum exhibited 7.1-fold higher odds of pregnancy than those presenting only follicles (p<0.05). The effect of corpus luteum presence was not evident in estrus detection (Figure 2a) but was clearly pronounced in positive pregnancy outcomes (Figure 2b).

**Table 3:** Effect of categorical variables on reproductive efficiency parameters in Creole cows from the High-Andean region subjected to FTAI protocols.

Categorical variables	Estrus		Pregnancy	
	Coef.	Sig.	Coef.	Sig.
FTAI Protocol (Protocol 2)	-0.34	0.75	-0.40	0.67
Age (>5 years-old)	-0.40	0.85	0.53	0.68
Number of calving (>2 calvings)	-1.24	0.55	-1.07	0.43
Body score condition	2.25	0.45	-3.33	0.15
Corpus luteum (Presence)	0.65	0.58	1.96	0.03
Straws breed (Fleckvieh)			-1.07	0.17

The coefficients and significances of the factors included in the logistic regression model are shown, taking as a reference the first category of each categorical variable. FTAI = Fixed-Time Artificial Insemination.

**Figure 2:**
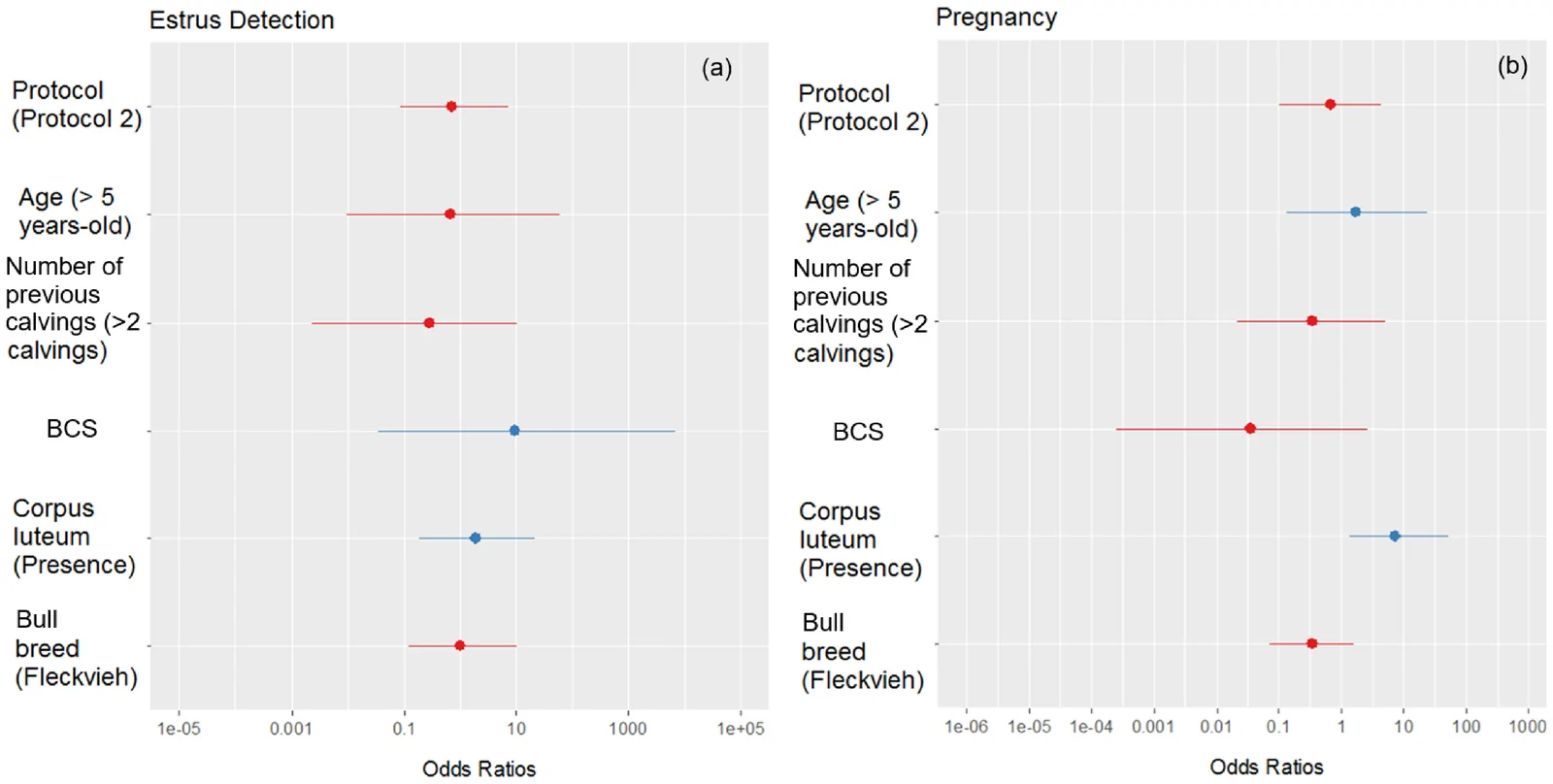
Odds ratios (OR) of categorical variables for estrus detection and pregnancy in Creole cows from the High-Andean region subjected to FTAI protocols. The ORs are shown as points and the 95% confidence intervals as side lines, where the red ones showed negative coefficients and the blue ones showed positive coefficients in the logistic regression.

The associations between (a) estrus detection success, (b) pregnancy, and calf sex according to categorical variables were analyzed using a χ^2^ test of independence (p<0.05). BCS = Body Condition Score.

Regarding cost comparisons, the three-handling T1 protocol incurred a baseline cost of USD 26.52 per animal, whereas the four-handling T2 protocol cost USD 26.24 per animal. This marginal variation was primarly driven by the estrogen sources utilized: Sincro CP® cost USD 0.58 per milliliter, while Sincrodiol® cost USD 0.31 per milliliter. Based on these imputs, the cost per pregnant cow for each treatment was determined by dividing the total cost of all inseminated cows by the total number of confirmed pregnancies. Consequently, the cost per pregnant cow was USD 46.41 for T1 and USD 46.86 for T2. Therefore, while T1 required a slightly higher initial investment per animal than T2, it ultimately yielded a lower cost per pregnant cow (Table 4). Furthermore, T1 offers the practical advantage of eliminating an entire day of animal handling required in T2, thereby reducing hidden expenses such as professional services and logistics/transportation.

**Table 4:** Comparison of costs per inseminated cow and pregnant cow according to FTAI protocol in Creole cows from the High-Andean region.

FTAI Protocols	Number of inseminated cows	Cost per inseminated cow (USD)	Number of pregnant cows	Cost per pregnant cow (USD)
T1 (three-handling)	35	26.52	20	46.41
T2 (four-handling)	25	26.24	14	46.86

FTAI = Fixed-Time Artificial Insemination.

## Discussion

In this study, the reproductive efficiency of Creole cows under High-Andean conditions was evaluated using two FTAI protocols. The results demostrate that FTAI utilizing progesterone BID devices with the T1 (three-handling) or T2 (four-handling) protocol produced comparable pregnancy rates within this experimental group (57.1% and 56.0%, respectively). Previous studies suggest that Creole cows may exhibit a unique response to hormonal protocols standardized for specialized breeds; this variation is often attributed to differences in ovarian function, follicular wave dynamics, growth rates, and preovulatory follicle diameter (Pérez-Ruiz et al., 2022; Zárate et al., 2010). Specifically, Creole cows display lower follicular growth rates and predominantly exhibit two follicular waves per estrous cycle, in contrast to the three waves typically observed in specialized breeds, which may result in earlier ovulations by an average of 6.2 hours (Quezada-Casasola et al., 2013, 2015). Consequently, advancing the timing of FTAI to approximately 28 hours after the onset of estrus has been suggested for this biotype (Quezada-Casasola et al., 2013). Nevertheless, the pregnancy rates acchieved in the present study fall well within the 50-70% success range reported for synchronized cattle subjected to progestogen-based FTAI (Marizancén & Artunduaga, 2017). Although pregnancy rates were statistically similar between treatments, the numerically higher estrus synchronization rate observed in T1 may be attributed to the longer follicular development period (8 days vs. 7 days in T2) prior to device removal, which likely favored the growth of the dominant and preovulatory follicle.

The duration of progesterone device exposure directly influences follicular development before the induction of the preovulatory LH surge. Physiologically, prolonged progesterone exposure suppresses LH pulsatility through negative feedback on the hypothalamic-pituitary axis, allowing the dominant follicle more time to develop and produce estradiol. Upon device removal, the cessation of this inhibition promotes high-frequency LH pulses that drive final follicular maturation and estradiol secretion; this triggers the GnRH/LH surge required for ovulation via positive feedback on the hypothalamus (Bó et al., 2018; Macedo et al., 2021). Larger preovulatory follicles secrete greater amounts of estradiol, which is strongly associated with enhanced oocyte competence, higher fertilization rates, and superior embryonic development. This physiological sequence may explain why the 8-day protocol (T1) tended to yield a higher estrus detection rate (88.6%) than the 7-day protocol (T2; 80.0%), as the additional day of progesterone priming may have promoted the development of larger, more synchronized preovulatory follicles. Coversely, in Caqueteño Creole cattle, a 100% estrus response with larger follicular diameters was reported alongside a 40% pregnancy rate using estradiol benzoate on day 9, compared to a 33.3% pregnancy rate using estradiol cypionate on day 8 (Quijano et al., 2015), suggesting that the timing of progesterone device removal and the choice of estrogen source interact to influence outcomes.

Similar pregnancy rates have been reported across diverse cattle populations. For instance, Torquati et al. (2011) reported pregnancy rates of 53.0% with estradiol cypionate (ECP) and 48.2% with estradiol benzoate (EB) in Angus cows, whereas lower rates (33.3% for ECP and 40.0% for EB) were observed in protocols utilizing an 8-day device removal (Quijano et al., 2015). In contrast, Morales and Cavestany (2012) reported pregnancy rates ranging from 64.0% to 70.0% in beef cows using a four-handling protocol. In Rarámuri Creole cows managed under a four-handling protocol (CIDR + EB) and inseminated at 36-43 hours, a 100% estrus response was achieved, but the pregnancy rate was only 9.09%, possibly due to early estrus expression and delayed FTAI scheduling (Zárate et al., 2010). Lower success rates have also been reported in Brahman (*Bos indicus*) cows using ECP (51.0%) versus EB (30.0%) (Peralta et al., 2010).

Although pregnancy rates are comparable between FTAI protocols utilizing ECP (three handlings) and EB (four handlings), contemporary reproductive emphasizes optimizing overall operational efficiency by minimizing animal handling and eliminating the need for estrus detection (Pérez-Ruiz et al., 2022). Furthermore, the use of EB faces increasing regulatory restrictions in some countries due to concerns regarding hormone residues in food products and potential estrogenic risk to human health, which has driven to adoption of analogs such as ECP (Bó et al., 2016). While EB and ECP exert similar physiological effects on the dominant follicle and the corpus luteum, they differ in their pharmacokinetic profiles. Estradiol benzoate undergoes rapid hydrolysis with a short half-life of 12-24 hours, making it suitable for four-handling protocols. In contrast, ECP exhibits a prolonged half-life of 36-48 hours, making it highly appropriate for three-handling protocols. Replacing EB with ECP at the time of progesterone device removal simplifies herd management by safely reducing the required animal handlings from four to three without compromising efficacy (Colazo et al., 2003).

At the onset of the protocol, progesterone device insertion mimics luteal function, while the administration of EB induces the regression of any pre-existing dominant follicle and the atresia of remaining follicles, thereby inducing a new follicular wave. Upon device removal, both EB and ECP exert positive feedback that triggers GnRH release, increased LH pulsatility, and subsequent ovulation (Lucy et al., 2004; Martínez et al., 2005; Peralta et al., 2010). In this context, administering eCG at device removal further supports follicular development by stimulating dominant follicle growth and steroidogenic enzyme expression. This enhances LH pulse frequency, promotes ovulation, and increase both corpus luteum diameter and subsequent progesterone production (Núñez-Olivera et al., 2020; Pérez-Ruiz et al., 2022).

The administration of eCG may be particularly relevant under High-Andean conditions, where cattle frequently experience subclinical nutritional stress and anestrus exacerbated by hypobaric hypoxia. Hypobaric hypoxia at altitudes exceeding 3,500 meters above sea level has been shown to suppress neuroendocrine function by reducing LH pulsatility and impairing preovulatory follicle development, thereby compromising overall reproductive efficiency (Kumari et al., 2023). Because eCG functions as an exogenous gonadotropin possessing dual FSH- and LH-like activity, it effectively stimulates dominant follicle growth, upregulates steroidogenic enzymes in granulosa and theca cells, and enhances post-ovulatory luteal function via elevated progesterone secretion (Baruselli et al., 2023; Uddin et al., 2023). These combined effects are especially critical for anestrous or nutritionally compromised cows, as eCG helps overcome the suppressed hypothalamic-pituitary-ovarian axis characteristic of these challenging environments.

When exploring the influence of herd- and animal-related factors on reproductive efficiency, location (district), age, parity, body condition score, and bull breed exerted negligible effects, whereas the presence of a corpus luteum at the start of the protocol increased the odds of pregnancy (OR = 7.1) without influencing estrus expression. In dairy herds, reproductive outcomes are heavily dictated by body condition loss, energy/mineral balance, genetics, and animal comfort (Cardoso et al., 2021; Marizancén & Artunduaga, 2017) alongside factors such as parity, milk yield, semen quality, and technician skill (Diskin & Kenny, 2016; Rearte et al., 2018; Vasconcelos, 2017). Similarly, Müller-Sepúlveda et al. (2020) identified herd location, stocking density (>30 cows), and inseminator experience (1-4 years versus >10 years) as key drivers of pregnancy success in cattle. In High-Andean Creole cows, their rustic adaptability may mask the impact of some of these conventional variables due to lifelong exposure to environmental stressors; however, the presence of a functional corpus luteum at the start of the protocol remains a critical determinant of fertility.

A corpus luteum at the onset of synchronization indicates a cyclic cow capable of responding optimally to hormonal stimulation, ensuring greater physiological homogeneity within the group. Conversely, cows presenting only follicles are more likely to be in deep anestrous, respond poorly to synchronization, or experience ovulatory failure from persistent follicles. Furthermore, variations in circulating progesterone concentrations at the time of ECP or EB administration are heavily influenced by the presence of a corpus luteum; while this does not affect behavioral estrus, it is heavily linked to pregnancy success (Bó et al., 2002). Adequate post-ovulatory corpus luteum function is essential for creating a uterine environment conducive to the maternal recognition of the embryo (Pérez-Ruiz et al., 2022). Progesterone secreted by the corpus luteum acts on the endometrium to promote uterine receptivity by regulating endometrial gene expression, histotroph composition, and the secretion of molecules required for conceptus elongation and implantation (Forde & Lonergan, 2020). Moreover, robust progesterone concentrations during the first week post-ovulation are essential to stimulate the elongating embryo to synthesize interferon-tau (IFN-τ). This embryonic signal prevents endometrial prostaglandin PGF2α secretion, thereby blocking luteolysis and securing pregnancy maintenance (Wiltbank et al., 2022). Thus, cows with a functional corpus luteum at the start of synchronization are not only respond more responsive to the hormonal protocol but also provide a superior endocrine environment for embryo survival.

Finally, it should be noted that the sample size utilized in this study may have limited the statistical power to detect subtle differences in pregnancy rate between the two treatments. Consequently, the absence of statistically significant differences between T1 and T2 should be interpreted with caution. However, even in the absence of statistical variance, these protocols diverge significantly in terms of operational and logistical costs. Under the extensive production conditions characteristic of the High-Andean region, where remote accessibility and rugged terrain pose severe constraints, the three-handling protocol (T1) emerges as a technically and economically more efficient alternative to the four-handling protocol (T2).

## Conclusion

Both FTAI protocols evaluated demonstrated comparable effectiveness regarding estrus detection, pregnancy rates, and direct costs in High-Andean Creole cows, indicating that either approach can be successfully implemented. However, when accounting for total operational costs, labor requirements, and field feasibility, the three-handling protocol utilizing estradiol cypionate (T1) is highly recommended as the most practical and efficient reproductive management strategy for High-Andean cattle production systems.
